# Validation of a Chinese Version of the Parental Burnout Assessment

**DOI:** 10.3389/fpsyg.2020.00321

**Published:** 2020-03-13

**Authors:** Huabin Cheng, Wei Wang, Shengnan Wang, Yimin Li, Xia Liu, Yongxin Li

**Affiliations:** ^1^Institute of Psychology and Behavior, Henan University, Kaifeng, China; ^2^Institute of Developmental Psychology, Beijing Normal University, Beijing, China

**Keywords:** parental burnout, job burnout, parental burnout assessment, reliability, validity

## Abstract

Parenting is a meaningful endeavor but it also induces stress, which can cause parental burnout. In China, the assessment and study of parental burnout are still in their formative stages. To contribute to advancing this field, the present study aimed to develop and validate a Chinese version of the Parental Burnout Assessment (PBA). Questionnaires were distributed to 614 families (comprising students in the eighth grade and both of their parents; one questionnaire for each person) on two separate occasions (Time 1 and Time 2). The students were asked to self-report their loneliness and life satisfaction at Time 1 and their anti-social behavior at Time 2. Meanwhile, parents were asked to self-report their parental burnout and job burnout at both Time 1 and Time 2, their marriage satisfaction at Time 1, and their levels of agreeableness and neuroticism at Time 2. Using the data obtained, we performed exploratory and confirmatory factor analyses, which indicated that this version of the PBA had a single-factor structure. The α of the PBA was 0.938 at Time 1 and 0.952 at Time 2. At Time 1, parental burnout was positively related to their job burnout in emotional exhaustion and depersonalization, and negatively related to their marriage satisfaction. In addition, parental burnout was positively related to students’ life satisfaction, and mothers’ parental burnout was positively related to students’ loneliness. At Time 2, parental burnout showed positive relations with neuroticism and job burnout in emotional exhaustion and depersonalization, and was negatively related to agreeableness. In addition, mothers’ parental burnout was positively related to students’ anti-social behavior. Furthermore, parental burnout at Time 1 also showed positive relations with job burnout through emotional exhaustion and depersonalization measured at Time 2, and parental burnout at Time 1 was positively related to students’ anti-social behavior at Time 2. Overall, the present study confirmed the reliability and validity of the Chinese version of the PBA.

## Introduction

There has recently been considerable interest in parental burnout, and an increasing number of studies have been conducted in different countries, including the Netherlands (Van Bakel et al., 2018), Turkey (Demirhan et al., 2011), Iran (Beheshtipour et al., 2016), and India (Vinayak and Rohin Kaur, 2017). However, a recent review by Sánchez-Rodríguez et al. (2019) reported that although 39 theses regarding parental burnout were published in 1989–2018, none were conducted in China or used a Chinese sample. Parental quality and adolescents’ healthy growth are common concerns worldwide; therefore, it is necessary to investigate parental burnout in China, as it comprises 1/5 of the worldwide population.

The importance of investigating parental burnout in China should not be underestimated for many reasons. First, Chinese parents may have a stronger urge to have children than Western parents (International Social Survey Programme, 2002). This means that they might be more likely to experience parental stress, and if they fail to cope with this stress, parental burnout may occur. Second, traditional parenting practices continue to play an important role in China. For instance, the traditional perception of “men work outside; women perform housework inside” prevails in many Chinese families, which means that parenting is a mother’s duty. Meanwhile, the female labor force is encouraged to get into workplace, and Chinese women are starting to have a generally higher employment rate than women in Western countries (Dasgupta et al., 2015). Chinese career women must address both workplace and parenting stress with limited time and energy, which could lead to job or parental burnout or, even worse, both simultaneously. It is urgent that we carry out the study of parental burnout in China, and the first step should be the development of a measure with high reliability and validity.

Children are important components of marriage and family life. The birth of a child can bring happiness to the parents and the family in general, and give a new meaning to their lives. However, parenting responsibilities and activities can also represent new burdens and stress for the parents. In fact, studies have indicated that, like job burnout, which results from exposure to excessive job stress, parents who are exposed to chronic parental stress may experience parental burnout (Norberg, 2007, 2010; Lindström et al., 2011; Norberg et al., 2014). Considering this, Roskam et al. (2017) developed and tested a tool for measuring parental burnout, and found that 2–12% of parents in Belgium experienced such burnout. Building on this work, Van Bakel et al. (2018) reported that in the Netherlands, this rate was 0.2–7.7%, while Kawamoto et al. (2018) reported that it was 4.2–17.3% in Japan.

Parental burnout may have serious negative effects on three different levels. First, parental burnout may cause negative consequences for the parents themselves; for instance, parental burnout has been found to be positively related to suicidal and escape ideation, addiction, and sleep problems. Second, parental burnout may, for instance, increase conflicts with and estrangement toward partners or spouses. Third, the effects of parental burnout may impact children, as the risk of neglectful and violent behavior toward them may increase (Mikolajczak et al., 2018). In addition, Mikolajczak et al. (2019) provided further evidence that parental burnout increased the parents’ escape ideation, negligent behavior, and violence longitudinally.

In order to explore the incidence of parental burnout and its negative effects, the measurements were developed. Early studies on this topic used the existent job-burnout scale to assess parental burnout, by simply changing the words “service subject” to “baby” or “parenting work.” For instance, the Maslach Burnout Inventory (MBI; Maslach and Jackson, 1981), the leading measure of burnout, was firstly used by Pelsma (1989). Subsequently, several different versions of job-burnout scales were applied to assess parental burnout, such as the MBI-Human Services Survey (Demirhan et al., 2011; Jaramillo et al., 2016; Vinayak and Rohin Kaur, 2017) and the Shirom-Melma Burnout Questionnaire (Norberg, 2007; Lindström et al., 2011; Norberg et al., 2014; Beheshtipour et al., 2016).

The job burnout inventory (e.g., MBI) used to measure parental burnout may not provide adequate information regarding the validity and specificity of the parental burnout construct. In addition, some MBI items (e.g., “I feel like I’m at the end of my rope”) do not specifically indicate occupational work, which may confuse the boundary between parental and job burnout, especially for parents who perform both duties. Based on these arguments, Roskam et al. (2017) developed the Parental Burnout Inventory (PBI) and indicated that parental burnout encompasses three dimensions: overwhelming exhaustion related to one’s parental role, emotional distancing from one’s children, and a sense of ineffectiveness in the parental role.

Furthermore, the structure and content of the parental and job burnout constructs may differ on both a theoretical and practical level. For instance, because parents cannot “dehumanize” their children, depersonalization (a component of job burnout measured by the MBI) may not be suitable in the parenting context (Roskam et al., 2018). Consequently, adopting an inductive approach, Roskam et al. (2018) advanced the measurement of parental burnout by developing the Parental Burnout Assessment (PBA), which was designed to better represent the parental burnout construct. The PBA comprises four factors: “exhaustion in one’s parental role,” “contrast with previous parental self,” “feeling fed up with one’s parental role,” and “emotional distancing from one’s children.”

While the PBI and PBA are highly correlated, Roskam et al. (2018) argued that the PBA had four advantages over the PBI. First, the PBA contained the factor “contrast with previous parental self,” which was an inherent notion of burnout not measured in the PBI. Second, the PBI contained a reverse factor (i.e., a higher score denoted lower burnout symptoms), which may lead to confusion in practical applications. In contrast, scoring in the PBA was not reversed, thus assessing parental burnout more clearly. Third, in the parental tasks, the loss of efficacy may precede the loss of pleasure and fulfillment. Therefore, the factor “parental accomplishment and efficacy” in the PBI, which combined loss of parental accomplishment and efficacy together, may be inappropriate. Fourth, the PBA was free to use while the PBI was not. Based on these advantages, for the present study, we chose to translate the PBA into Chinese. Consistent with Roskam et al. (2018), the present study hypothesized that the Chinese version of PBA also contains four factors, and the factor structure would be the same between fathers’ and mothers’ responses.

In order to examine the external validity of the PBA in China, relevant variables associated with parental burnout were measured. First, parental burnout may correlate with parents’ personal characteristics. In line with prior studies, personality traits, including neuroticism and agreeableness (Le Vigouroux et al., 2017), and job burnout (Roskam et al., 2018) were assessed. Second, as noted by Mikolajczak et al. (2018, 2019), parental burnout is closely related to marital conflicts and partner-estrangement mindsets, which may induce dissatisfaction with marriage. Thus, marriage satisfaction was measured as well. Third, parental burnout may not only affect parents or their partners but also have negative effects on their children (Mikolajczak et al., 2018). Consequently, children’s loneliness, life satisfaction, and anti-social behavior were also measured in the present study.

In total, parents’ job burnout, marriage satisfaction, and personality traits (agreeableness and neuroticism), and children’s loneliness, life satisfaction, and anti-social behavior were measured. Specifically, concurrent validity was examined via cross-section correlation analysis, including the relationships between parental and job burnout, marriage satisfaction, agreeableness, and neuroticism, as well as the relationship between parental burnout and the children’s loneliness, life satisfaction, and anti-social behavior. Furthermore, predictive validity was examined by a regression analysis that was conducted with longitudinal data to examine the relationships between parental and job burnout, and parental burnout and children’s anti-social behavior.

## Method and Participants

To test the validity of the Chinese version of the PBA, data were collected from a sample of eighth-grade students (from a middle school located in an urban area of Kaifeng city, Henan Province) and their parents on two separate occasions (Time 1 and Time 2). Envelopes containing three questionnaires (one for the student and one for each parent) were distributed to 614 students during a class. They were asked to complete the questionnaire at home and bring the completed forms back to school. A month later, data were collected from the same sample in a similar fashion.

The final sample used for analysis comprised, at Time 1, 597 students, 458 fathers, and 531 mothers. The student sample comprised 307 males and 290 females, and the average age was 13.9 years (*SD* = 0.63). Fathers’ average age was 41.9 years (*SD* = 4.18) and mothers’ average age was 40.9 (*SD* = 4.09). At Time 2, 567 students, 312 fathers, and 329 mothers were examined.

### Measures

Except for the PBA, Chinese versions were available for all other questionnaire tools used, and each measurement had previously been determined to have satisfactory reliability and validity. At Time 1, PBA, parents’ job burnout, and marriage satisfaction were answered by parents; students’ loneliness and life satisfaction were answered by children. At Time 2, PBA, parents’ job burnout, and personality traits (agreeableness and neuroticism) were answered by parents; students’ anti-social behavior was answered by children. The example of items introduced below were in their original English version.

#### Parental Burnout

Parental burnout was measured using Roskam et al.’s (2018) 23-item PBA. This tool was administered at both Time 1 and Time 2. We sent an email to the author who developed the original version of the PBA and obtained permission to use and translate it. Three psychology researchers who were native speakers of Chinese and who had experience studying overseas translated these items into Chinese and made slight modifications to adapt the tool to the Chinese culture and context. Then, a native-English speaker researcher who was unfamiliar with the questionnaire back-translated the scale into English. The translated questionnaire was compared with the primary PBA to ensure that the meanings of the sentences were the same. The Chinese translation of the questionnaire was then distributed to 30 graduate students to evaluate how natural-sounding the sentences were. They were asked to evaluate naturalness using a five-point Likert scale, ranging from 1 (“not natural”) to 5 (“natural”). Based on the graduate students’ feedback, we made adjustments and then included the finalized items in the final scale that was distributed to the participants.

The final version of the questionnaire contained 23 items (e.g., “I feel as though I’ve lost my direction as a dad/mum”), and the items were rated using a seven-point Likert scale, ranging from 1 (“completely inconsistent”) to 7 (“completely consistent”). In line with the original version of the PBA, a higher score represented a higher number of burnout symptoms.

#### Job Burnout

Job burnout was assessed using the Chinese version of the MBI, which had previously been determined to have adequate reliability and validity for Chinese samples (Li, 2003). The Chinese version of the MBI encompassed three factors, each including five items: emotional exhaustion (e.g., “I feel burned out from my work”), depersonalization (e.g., “I don’t really care what happens to some recipients”), and reduced personal accomplishment (e.g., “I can easily create a relaxed atmosphere with my recipients”). Items were rated using a seven-point Likert scale, ranging from 1 (“completely inconsistent”) to 7 (“completely consistent”), with a higher score representing a higher burnout. At Time 1, the Cronbach’s α values for the fathers’ responses were 0.772 for emotional exhaustion (ω = 0.775), 0.712 for depersonalization (ω = 0.714), and 0.733 for reduced personal accomplishment (ω = 0.726). The Cronbach’s α values for mothers’ responses were.737 for emotional exhaustion (ω = 0.752), 0.710 for depersonalization (ω = 0.775), and 0.674 for reduced personal accomplishment (ω = 0.675). At Time 2, the Cronbach’s α values for the fathers’ responses were 0.766 for emotional exhaustion (ω = 0.768), 0.809 for depersonalization (ω = 0.811), and 0.763 for reduced personal accomplishment (ω = 0.763), while those for the mothers’ responses were 0.731 for emotional exhaustion (ω = 0.735), 0.771 for depersonalization (ω = 0.776), and 0.752 for reduced personal accomplishment (ω = 0.754).

#### Parents’ Marriage Satisfaction

Parents’ marriage satisfaction was assessed using the Chinese version of the Marriage Satisfaction Scale, a subscale of the ENRICH Marital inventory (Olson et al., 1987; Wang et al., 1999). The Marriage Satisfaction Scale contained 10 items, including “my partner and I understand each other perfectly.” These items were rated using a five-point Likert scale ranging from 1 (“strongly disagree”) to 5 (“strongly agree”). This scale included five reverse scoring items, and a higher score represented higher marriage satisfaction. In the present study, the Cronbach’s α values were 0.819 for fathers and 0.850 for mothers, the McDonald’s ω was 0.823 for fathers and 0.852 for mothers.

#### Parents’ Agreeableness and Neuroticism

Parents’ agreeableness and neuroticism were assessed using the Agreeableness and Neuroticism factors of the Chinese version of the Big Five Personality Traits Scale (Luo and Dai, 2015). Each factor had five items; for example, “distrustful–trustful” for agreeableness and “nervous–at ease” for neuroticism. Items were rated using a six-point bipolar adjective scale. The response scale ranged from 1 (“full endorsement of neuroticism/full endorsement of disagreeableness”) to 6 (“full endorsement of emotional stability/full endorsement of agreeableness”), and a higher score meant higher emotional stability or agreeableness. In the present study, for fathers, the Cronbach’s α values were 0.865 for agreeableness (ω = 0.865) and 0.829 for neuroticism (ω = 0.834), while for mothers, the Cronbach’s α values were 0.832 for agreeableness (ω = 0.833) and 0.764 for neuroticism (ω = 0.768).

#### Students’ Life Satisfaction

Students’ life satisfaction was measured with the Adolescent Life Satisfaction Scale (Zhang et al., 2004), which contained 36 items (e.g., “I like being with my parents”). The items were rated using a seven-point Likert scale, ranging from 1 (“completely inconsistent”) to 7 (“completely consistent”). This scale included four reverse scoring items, and a higher score meant a higher life satisfaction. The Cronbach’s α value was 0.948 and the McDonald’s ω was 0.941.

#### Students’ Loneliness

Students’ loneliness was measured with the Chinese version of the UCLA Loneliness Scale (Wang, 1995), which contained 18 items (e.g., “I lack companionship”). The items were rated using a four-point Likert scale, ranging from 1 (“I never feel this way”) to 4 (“I often feel this way”). This scale included eight reverse scoring items, and a higher score represented a higher level of loneliness. For the present study, the Cronbach’s α value was 0.891 and the McDonald’s ω was 0.984.

#### Students’ Anti-social Behavior

Students’ anti-social behavior was measured using the Adolescent Anti-Social Behavior Scale (Achenbach and Edelbrock, 1987), which had well-established reliability and validity across cultures (Barber et al., 2005). The scale contained nine items; for example, “I hang around with kids who get in trouble.” The items were rated using a four-point Likert scale, ranging from 1 (“never”) to 4 (“always”), and a higher score represented more anti-social behavior. For the present study, the Cronbach’s α value was 0.802 and the McDonald’s ω was 0.804.

#### Other Measures

Demographic items, including students’ gender and age, and parents’ age and occupation, were recorded.

## Results

### Data Analysis

Before conducting a factor analysis, all the questionnaires including missing data were removed from the sample. They were removed because either most of the questions were unanswered or the questionnaire was blank. Furthermore, the Welch’s test was conducted between the removed and kept data groups. There were no significant differences between the children’s age (*t* = −0.44, *df* = 266.19, *p* = 0.66), gender (*t* = 0.28, *df* = 402.86, *p* = 0.78), and the parents’ age (for fathers: *t* = 0.60, *df* = 1.00, *p* = 0.66; for mothers: *t* = −0.08, *df* = 6.01, *p* = 0.94). These results suggested that removing the incomplete questionnaires did not bias our results.

In order to examine the constructive validity and reliability of the Chinese version of the PBA, exploratory factor analysis (EFA) was conducted on the data collected at Time 1, and confirmatory factor analysis (CFA; estimated by Maximum Likelihood Method) was conducted on the data collected at Time 2. Furthermore, the gender invariance was examined by multi-group CFA. To examine the concurrent validity of the PBA, correlation analyses were conducted on parental burnout, job burnout, and marriage satisfaction, as well as on students’ loneliness and life satisfaction using the data collected at Time 1. Correlation analyses were conducted on parental burnout, job burnout, agreeableness, neuroticism, as well as on students’ anti-social behavior using the data collected at Time 2. To examine the predictive validity of the PBA, Time 1 and Time 2 data were combined. Regression analyses were conducted on parental burnout (Time 1) and job burnout (Time 2), and on students’ anti-social behavior (Time 2). These analyses were conducted using R 3.6.1 for Windows.

### Factor Analysis and Correlation Analysis for Data Collected at Time 1

To ensure we had an adequate sample size for CFA, we combined fathers’ and mothers’ parental burnout data. Thus, for Time 1, the sample size was 989, which satisfied Hinkin’s (1998) recommendation regarding scale development. Based on the practice of Roskam et al. (2018), CFA was conducted. First, the 23 items were loaded onto the four original factors, and the four factors were allowed to be correlated. However, the model did not show a good fit [χ^2^ = 1906.53, *df* = 231, *p* < 0.001, CFI = 0.547, root mean square error of approximation (RMSEA) = 0.143, standardized root mean square residual (SRMR) = 0.315]. Then, a higher-order model was examined: a second-order factor, general parental burnout factor with four lower-order factors, the model fit did not improve significantly [Δχ2(6) = 169.23, *p* < 0.001]. These results suggested that the four-factor structure was not supported by the present data and that the factor structure differed between the European and Chinese samples (this will be discussed in the section “Discussion”). Therefore, in order to identify the factor structure of the Chinese version of the PBA, EFA was conducted.

In order to identify the factor structure of the Chinese version of the PBA, the EFA was conducted. Parallel analysis and the minimum average partial (MAP) method were conducted on the 23 items to determine the number of factors in the Chinese version of the PBA. The results suggested a two-factor structure. When a two-factor structure was set (through maximum likelihood estimation and Promax rotation), two items with factor loadings under 0.40 (i.e., “I’m so tired out by my role as a parent that sleeping doesn’t seem like enough” and “my role as a parent uses up all my resources”) were removed. Then, parallel analysis and the MAP method were again conducted on 21 items.

The result of the parallel analysis suggested a two-factor structure, while the MAP method suggested a single-factor structure. Parallel analysis can overestimate the number of factors, while the MAP method can underestimate them (Hori, 2005). However, when parallel analysis and the MAP method recommend the same number, the result may be considered robust. If the numbers differ, factor analysis should be conducted, beginning with the recommendations of the parallel analysis and decreasing the number of factors until the factor structure is interpretable, or by beginning with the recommendations of the MAP method and increasing the number of factors until the factor structure is interpretable. Once a two-factor structure was set (again through maximum likelihood estimation and Promax rotation), only three items loaded on the second factor, while all others loaded on the first factor. It was consequently difficult to explain the two-factor structure and name the factors; therefore, we combined them into a single factor. When the single-factor structure was set, all 21 items showed factor loadings of over 0.40. This single factor explained 45.07% of the variance, and the Cronbach’s α value of the factor was 0.938, thereby meeting Nunnaly’s (1978) criterion. In addition, when the data for the fathers and mothers were separated, the results also supported the single-factor structure. The results of the factor analysis are shown in Table 1.

**TABLE 1:** Results of the exploratory factor analysis (maximum likelihood estimation and Promax rotation).

**Item**	**Combined fathers with mothers**	**Fathers’ answer**	**Mothers’ answer**
	**α = 0.938**	**α = 0.916**	**α = 0.927**
	**ω = 0.938**	**ω = 0.916**	**ω = 0.927**
15. I feel as though I’ve lost my direction as a dad/mum	**0.486**	**0.490**	**0.508**
1. I feel completely run down by my role as a parent	**0.587**	**0.563**	**0.616**
6. I have zero energy for looking after my child(ren)	**0.670**	**0.642**	**0.714**
10. I don’t think I’m the good father/mother that I used to be to my child(ren)	**0.675**	**0.675**	**0.682**
16. I can’t stand my role as father/mother any more	**0.715**	**0.745**	**0.721**
18. I feel like I can’t take any more as a parent	**0.688**	**0.747**	**0.710**
8. I sometimes have the impression that I’m looking after my child(ren) on autopilot	**0.557**	**0.607**	**0.561**
2. I have the sense that I’m really worn out as a parent	**0.661**	**0.641**	**0.708**
4. When I get up in the morning and have to face another day with my child(ren), I feel exhausted before I’ve even started	**0.685**	**0.731**	**0.706**
20. I don’t enjoy being with my child(ren)	**0.612**	**0.692**	**0.618**
19. I feel like I can’t cope as a parent	**0.784**	**0.813**	**0.798**
11. I tell myself that I’m no longer the parent I used to be	**0.754**	**0.743**	**0.788**
21. I do what I’m supposed to do for my child(ren), but nothing more	**0.494**	**0.545**	**0.491**
17. I can’t take being a parent any more	**0.659**	**0.681**	**0.696**
12. I’m ashamed of the parent that I’ve become	**0.658**	**0.660**	**0.670**
13. I’m no longer proud of myself as a parent	**0.650**	**0.703**	**0.674**
14. I have the impression that I’m not myself any more when I’m interacting with my child(ren)	**0.641**	**0.674**	**0.641**
23. I’m no longer able to show my child(ren) how much I love them	**0.725**	**0.709**	**0.750**
5. I find it exhausting just thinking of everything I have to do for my child(ren)	**0.682**	**0.751**	**0.668**
22. Outside the usual routines (lifts in the car, bedtime, meals), I’m no longer able to make an effort for my child(ren)	**0.623**	**0.637**	**0.666**
9. I’m in survival mode in my role as a parent	**0.580**	**0.599**	**0.623**
**3. I’m so tired out by my role as a parent that sleeping doesn’t seem like enough**	0.129	0.100	0.109
**7. My role as a parent uses up all my resources**	0.067	−0.108	0.097

*The numbers on the left represent the original order of the questions in the work of Roskam et al. (2018). Items 3 and 7 were deleted. Note: The bold values are factor loadings.*

In order to further test the validity of the Chinese version of the PBA, correlation analysis was conducted. When we checked the distribution of the data, parental burnout scores did not show a normal distribution. In accordance with a prior study (Mikolajczak et al., 2018), the PBA scores were normalized by log transformations (for fathers’ answer, skewness = 1.35 and kurtosis = 1.60; for mothers’ answer, skewness = 1.01 and kurtosis = 0.52; combined with fathers and mothers’ answer, skewness = 1.16 and kurtosis = 0.93). When the correlation analysis was conducted with the transformed scores, there were no significant differences between the results of the transformed scores and the original ones, so we reported the results of the original scores.

The results are shown in Table 2. Fathers’ parental burnout showed positive correlations with their emotional exhaustion (*r* = 0.385, *p* < 0.01), depersonalization (*r* = 0.312, *p* < 0.01), reduced personal accomplishment (*r* = 0.230, *p* < 0.01), and a negative correlation with their marriage satisfaction (*r* = −0.400, *p* < 0.01). Similarly, mothers’ parental burnout had positive correlations with their emotional exhaustion (*r* = 0.409, *p* < 0.01) and depersonalization (*r* = 0.226, *p* < 0.01), and a negative correlation with their marriage satisfaction (*r* = −0.309, *p* < 0.01). However, mothers’ parental burnout showed no significant correlation with reduced personal accomplishment (*r* = −0.056, *ns*). Meanwhile, mothers’ parental burnout showed positive correlations with the children’s loneliness (*r* = 0.168, *p* < 0.01), whereas fathers’ parental burnout showed no significant correlation with the children’s loneliness (*r* = 0.070, *ns*). Both fathers’ and mothers’ parental burnout were negatively correlated with students’ life satisfaction (for fathers, *r* = −0.163, *p* < 0.01; for mothers, *r* = −0.265, *p* < 0.01). In general, the Chinese version of the PBA showed good validity.

**TABLE 2:** Results of the correlation analysis with data collected at Time 1.

		***M***	***SD***	**①**	**②**	**③**	**④**	**⑤**	**⑥**	**⑦**	**⑧**	**⑨**	**⑩**	**⑪**	**⑫**
①	FPBA	1.75	0.82	(0.939)											
②	FEE	2.94	1.38	0.385**	(0.772)										
③	FDE	2.35	1.20	0.312**	0.556**	(0.712)									
④	FRPA	3.37	1.41	0.230**	0.177**	0.184**	(0.733)								
⑤	FMS	3.91	0.79	−0.400**	−0.391**	−0.274**	–0.097	(0.819)							
⑥	MPBA	1.94	0.91	0.494**	0.346**	0.211**	0.074	−0.219**	(0.940)						
⑦	MEE	3.02	1.38	0.289**	0.480**	0.287**	0.081	−0.238**	0.409**	(0.737)					
⑧	MDE	2.21	1.21	0.226**	0.241**	0.427**	0.170**	−0.208**	0.226**	0.451**	(0.710)				
⑨	MRPA	3.47	1.40	0.000	0.028	–0.049	0.149**	0.080	–0.056	0.073	–0.031	(0.674)			
⑩	MMS	3.71	0.90	−0.247**	−0.291**	−0.329**	–0.038	0.507**	−0.309**	−0.303**	−0.214**	0.193**	(0.850)		
⑪	SLO	1.84	0.52	0.070	0.124^∗^	0.082	0.005	−0.172**	0.168**	0.093	0.093	–0.051	−0.195**	(0.802)	
⑫	SLS	4.87	1.03	−0.163**	−0.210**	−0.182**	–0.006	0.247**	−0.265**	−0.154**	−0.119^∗^	0.121^∗^	0.236**	−0.665**	(0.891)

F, father; M, mother; S, student; PBA, parental burnout assessment; EE, emotional exhaustion; DE, depersonalization; RPA, reduced personal accomplishment; MS, marriage satisfaction; LO, loneliness; LS, life satisfaction. The Cronbach’s α values are shown in brackets. *p < 0.05; **p < 0.01.

### Factor Analysis and Correlation Analysis for Data Collected at Time 2

To further examine the reliability and validity of the Chinese version of the PBA, CFA and correlation analysis were conducted using data collected at Time 2. To ensure we had an adequate sample size for CFA, fathers’ and mothers’ responses were combined. When the 21 items were loaded onto a single factor, the result generally showed a good fit (χ^2^ = 1106.181, *df* = 166, *p* < 0.001, CFA = 0.902, RMSEA = 0.095, SRMR = 0.050). Meanwhile, the Cronbach’s α value for the factor was.953, which supported the single-factor structure. Furthermore, when separating fathers’ (α = 0.956) and mothers’ answers (α = 0.952) in conformational factor analyses, the single-factor structure was also supported. The results of the CFA are shown in Table 3.

**TABLE 3:** Results of the confirmatory factor analysis.

**Items**	**Combined fathers with mothers**	**Fathers’ answer**	**Mothers’ answer**
	**α = 0.952**	**α = 0.956**	**α = 0.952**
	**ω = 0.952**	**ω = 0.956**	**ω = 0.953**
15. I feel as though I’ve lost my direction as a dad/mum	**0.489**	**0.392**	**0.528**
1. I feel completely run down by my role as a parent	**0.592**	**0.606**	**0.615**
6. I have zero energy for looking after my child(ren)	**0.719**	**0.776**	**0.708**
10. I don’t think I’m the good father/mother that I used to be to my child(ren)	**0.644**	**0.671**	**0.683**
16. I can’t stand my role as father/mother any more	**0.767**	**0.822**	**0.773**
18. I feel like I can’t take any more as a parent	**0.729**	**0.749**	**0.728**
8. I sometimes have the impression that I’m looking after my child(ren) on autopilot	**0.678**	**0.551**	**0.696**
2. I have the sense that I’m really worn out as a parent	**0.770**	**0.754**	**0.743**
4. When I get up in the morning and have to face another day with my child(ren), I feel exhausted before I’ve even started	**0.768**	**0.795**	**0.695**
20. I don’t enjoy being with my child(ren)	**0.654**	**0.785**	**0.570**
19. I feel like I can’t cope as a parent	**0.714**	**0.538**	**0.856**
11. I tell myself that I’m no longer the parent I used to be	**0.785**	**0.774**	**0.773**
21. I do what I’m supposed to do for my child(ren), but nothing more	**0.558**	**0.656**	**0.531**
17. I can’t take being a parent any more	**0.778**	**0.873**	**0.765**
12. I’m ashamed of the parent that I’ve become	**0.730**	**0.618**	**0.761**
13. I’m no longer proud of myself as a parent	**0.751**	**0.766**	**0.797**
14. I have the impression that I’m not myself any more when I’m interacting with my child(ren)	**0.728**	**0.810**	**0.755**
23. I’m no longer able to show my child(ren) how much I love them	**0.738**	**0.631**	**0.765**
5. I find it exhausting just thinking of everything I have to do for my child(ren)	**0.804**	**0.838**	**0.768**
22. Outside the usual routines (lifts in the car, bedtime, meals), I’m no longer able to make an effort for my child(ren)	**0.686**	**0.694**	**0.736**
9. I’m in survival mode in my role as a parent	**0.663**	**0.609**	**0.689**
**3. I’m so tired out by my role as a parent that sleeping doesn’t seem like enough**	0.300	0.305	0.267
**7. My role as a parent uses up all my resources**	0.216	0.196	0.199

*The order numbers on the left are the original order numbers in the work of Roskam et al. (2018). Items 3 and 7 were deleted. Note: The bold values are factor loadings.*

In order to test the gender invariance of factor structure of the PBA, the multi-group CFA was conducted, in which the 21 items were loaded on a single factor. Two groups (mother and father) and three models were set. The three models included the unconstrained model, the constrained measurement weight as same, and the constrained structural covariances as same. Results showed that there were no significant differences between the unconstrained model and the constrained measurement weight model [Δχ^2^ (20) = 15.26, *p* = 0.761], which suggested that the factor loading was the same between the two groups. There were no significant differences between the unconstrained model and the constrained structural covariances model [Δχ^2^ (42) = 32.39, *p* = 0.857], which suggested that the factor covariances were the same between the two groups. In general, the factor structures were invariant between the two groups.

In order to further test the validity of the Chinese version of the PBA, correlation analyses were conducted. The results are shown in Table 4. Fathers’ parental burnout had positive correlations with fathers’ emotional exhaustion (*r* = 0.451, *p* < 0.01), depersonalization (*r* = 0.521, *p* < 0.01), and neuroticism (*r* = 0.443, *p* < 0.01), and a negative correlation with fathers’ agreeableness (*r* = −0.503, *p* < 0.01). Similarly, mothers’ parental burnout had positive correlations with mothers’ emotional exhaustion (*r* = 0.358, *p* < 0.01), depersonalization (*r* = 0.467, *p* < 0.01), and neuroticism (*r* = 0.403, *p* < 0.01), and negative correlation with mothers’ agreeableness (*r* = −0.359, *p* < 0.01). However, at Time 2, fathers’ parental burnout was not significantly correlated with reduced personal accomplishment (*r* = −0.092, *ns*), whereas mothers’ parental burnout showed a significant correlation with reduced personal accomplishment (*r* = −0.134, *ns*). Furthermore, mothers’ parental burnout showed positive correlations with the children’s anti-social behavior (*r* = 0.145, *p* < 0.01), whereas fathers’ parental burnout showed no significant correlation with the children’s anti-social behavior (*r* = 0.007, *ns*). Generally, these results further supported the validity of the Chinese version of the PBA.

**TABLE 4:** Results of the correlation analysis with data collected at Time 2.

		***M***	***SD***	**①**	**②**	**③**	**④**	**⑤**	**⑥**	**⑦**	**⑧**	**⑨**	**⑩**	**⑪**	**⑫**	**⑬**
①	FPBA	2.01	0.97	(0.956)												
②	FAG	4.67	1.02	−0.503**	(0.865)											
③	FNE	2.64	0.97	0.443**	−0.806**	(0.829)										
④	FEE	3.00	1.31	0.451**	−0.218**	0.282**	(0.766)									
⑤	FDE	2.55	1.33	0.521**	−0.371**	0.319**	0.612**	(0.809)								
⑥	FRPA	3.54	1.44	–0.092	0.293**	−0.209**	0.196**	–0.091	(0.763)							
⑦	MPBA	2.08	0.99	0.465**	−0.384**	0.359**	0.191**	0.228**	–0.110	(0.952)						
⑧	MAG	4.78	1.01	−0.353**	0.532**	−0.466**	−0.243**	−0.284**	0.143^∗^	−0.359**	(0.832)					
⑨	MNE	2.67	0.98	0.283**	−0.409**	0.449**	0.246**	0.223**	–0.111	0.403**	−0.738**	(0.764)				
⑩	MEE	3.07	1.29	0.331**	−0.196**	0.253**	0.376**	0.238**	–0.009	0.358**	−0.216**	0.353**	(0.731)			
⑪	MDE	2.34	1.25	0.380**	−0.383**	0.329**	0.312**	0.429**	–0.077	0.467**	−0.368**	0.278**	0.456**	(0.771)		
⑫	MRPA	3.61	1.46	–0.022	0.148^∗^	−0.155^∗^	–0.025	–0.082	0.310**	−0.134^∗^	0.214**	−0.170**	0.224**	–0.041	(0.752)	
⑬	SASB	1.86	0.30	0.007	0.086	–0.029	0.118^∗^	0.014	0.074	0.145^∗^	0.003	0.054	0.086	0.019	0.054	(0.716)

F, father; M, mother; S, student; PBA, parental burnout assessment; AG, agreeableness; NE, neuroticism; EE, emotional exhaustion; DE, depersonalization; RPA, reduced personal accomplishment; ASB, anti-social behavior. The Cronbach’s α values are shown in brackets. *p < 0.05; **p < 0.01.

### Correlation Analysis of the Data Collected at Time 1 and Time 2

To examine the predictive validity of the PBA, correlation and regression analyses were also conducted regarding parental burnout (measured at Time 1 and Time 2), parents’ job burnout (measured at Time 1 and Time 2), and students’ antisocial behavior (measured at Time 2). The results are shown in Table 5.

**TABLE 5:** Results of the correlation analysis with data collected at both Time 1 and Time 2.

		**①**	**②**	**③**	**④**	**⑤**	**⑥**	**⑦**	**⑧**	**⑨**	**⑩**	**⑪**	**⑫**	**⑬**	**⑭**	**⑮**	**⑯**
①	T1FPBA																
②	T1FEE	0.397**															
③	T1FDE	0.330**	0.565**														
④	T1FRPC	0.095	–0.062	0.036													
⑤	T1MPBA	0.443**	0.355**	0.242**	0.017												
⑥	T1MEE	0.277**	0.468**	0.332**	0.116^∗^	0.409**											
⑦	T1MDE	0.220**	0.241**	0.434**	0.146**	0.197**	0.463**										
⑧	T1MRPC	0.036	0.002	0.037	0.320**	0.076	–0.038	0.026									
⑨	T2FPBA	0.268**	0.302**	0.147^∗^	0.078	0.144^∗^	0.185**	0.258**	0.000								
⑩	T2FEE	0.322**	0.360**	0.300**	0.130^∗^	0.188**	0.190**	0.223**	0.047	0.460**							
⑪	T2FDE	0.268**	0.342**	0.347**	0.108	0.175**	0.140^∗^	0.178**	0.072	0.376**	0.599**						
⑫	T2FRPC	0.052	0.085	0.069	0.185**	0.082	0.088	0.022	0.117	0.059	−0.195**	0.103					
⑬	T2MPBA	0.130	0.170**	0.120	–0.011	0.411**	0.294**	0.198**	0.138^∗^	0.425**	0.268**	0.226**	0.099				
⑭	T2MEE	0.226**	0.259**	0.163**	0.052	0.260**	0.337**	0.171**	0.057	0.419**	0.401**	0.267**	0.007	0.373**			
⑮	T2MDE	0.150^∗^	0.210**	0.242**	0.114	0.240**	0.209**	0.304**	0.095	0.337**	0.312**	0.454**	0.094	0.439**	0.484**		
⑯	T2MRPC	–0.018	–0.016	0.113	0.309**	0.048	0.011	0.111^∗^	0.256**	–0.056	0.009	0.088	0.309**	0.084	−0.235**	0.023	
⑰	T2SASB	0.139^∗^	0.144**	0.069	–0.001	0.123^∗^	0.126^∗^	0.075	0.007	0.099	0.124^∗^	0.000	−0.120^∗^	0.168**	0.077	0.024	–0.044

T, time; F, father; M, mother; PBA, parental burnout assessment; EE, emotional exhaustion; DE, depersonalization; RPA, reduced personal accomplishment; ASB, anti-social behavior. *p < 0.05; **p < 0.01.

Fathers’ parental burnout at Time 1 showed positive correlations with their emotional exhaustion (*r* = 0.305, *p* < 0.01) and depersonalization (*r* = 0.279, *p* < 0.01) at Time 2. However, the relationship between fathers’ parental burnout at Time 1 and fathers’ reduced personal accomplishment at Time 2 was not significant (*r* = 0.036, n.s.). Similarly, mothers’ parental burnout at Time 1 showed a positive correlation with mothers’ emotional exhaustion (*r* = 0.271, *p* < 0.01) and depersonalization (*r* = 0.245, *p* < 0.01) at Time 2. However, the relationship between mothers’ parental burnout at Time 1 and mothers’ reduced personal accomplishment at Time 2 was not significant (*r* = 0.082, n.s.). Fathers’ emotional exhaustion and depersonalization at Time 1 showed positive correlations with fathers’ parental burnout at Time 2 (*r* = 0.360, *p* < 0.01; *r* = 0.210, *p* < 0.01). However, the relationship between fathers’ reduced personal accomplishment at Time 1 and fathers’ parental burnout at Time 2 was not significant (*r* = 0.082, n.s.). Similarly, mothers’ emotional exhaustion, depersonalization, and reduced personal accomplishment at Time 1 showed positive correlations with parental burnout at Time 2 (*r* = 0.337, *p* < 0.01; *r* = 0.209, *p* < 0.01; *r* = 0.138, *p* < 0.05). Both fathers’ and mothers’ parental burnout showed positive correlations with the children’s anti-social behavior (for fathers, *r* = 0.144, *p* < 0.01; for mothers, *r* = 0.123, *p* < 0.05).

**FIGURE 1:**
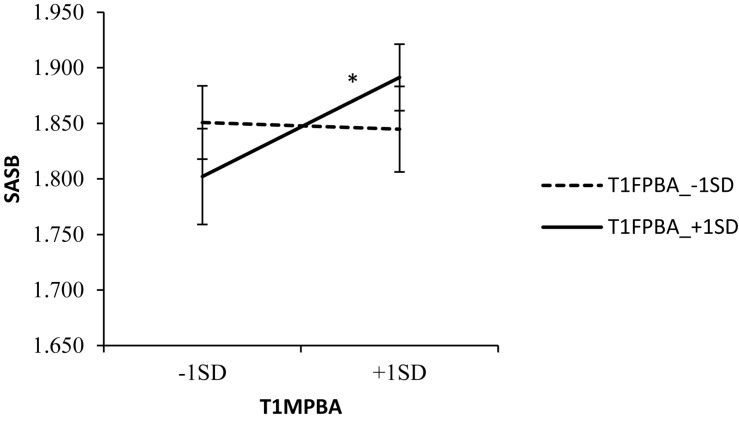
Interaction of fathers’ and mothers’ PBA at Time 1 with students’ anti-social behavior at Time 2 (SASB: Students’ anti-social behavior; T1MPBA: Time 1 mothers’ parental burnout).

In order to further examine the predictive validity of the Chinese version of the PBA, regression analyses were conducted. When age was controlled, fathers’ parental burnout at Time 1 showed a positive correlation with fathers’ emotional exhaustion (β = 0.322, *p* < 0.01; Δ*R*^2^ = 0.10) and depersonalization (β = 0.268, *p* < 0.01; Δ*R*^2^ = 0.07) at Time 2. Similarly, mothers’ parental burnout at Time 1 had a positive correlation with mothers’ emotional exhaustion (β = 0.260, *p* < 0.01; Δ*R*^2^ = 0.06) and depersonalization (β = 0.240, *p* < 0.01; Δ*R*^2^ = 0.06) at Time 2. Furthermore, at Time 1, the main effects of fathers’ parental burnout and mother’s parental burnout were not significant (β = 0.114, *n.s*.; β = 0.076, *n.s.*). However, their interaction was significant (β = 0.200, *p* < 0.01; Δ*R*^2^ = 0.029; *p* < 0.01), and a simple slope test was conducted. As shown in Figure 1, when fathers’ parental burnout was high, students’ anti-social behavior was higher with higher mother’s parental burnout. However, when fathers’ parental burnout was low, students’ anti-social behavior did not change even if the mothers’ parental burnout was high.

## Discussion

The present study aimed to explore the incidence of parental burnout in China, to investigate the relationship and mechanisms between parental burnout and other relevant variables, provide suggestions to prevent parental burnout, and improve parenting quality. To that end, the first step was to develop a highly reliable and valid scale to measure parental burnout. Based on the work of Roskam et al. (2018), through a longitudinal design and the use of family unit data, the present study developed a Chinese version of the PBA, and provided preliminary support for its reliability and validity.

Our results showed a different factor structure to that reported in the prior work of Roskam et al. (2018), in which a four-factor structure was supported. However, the four factors of the original PBA (Roskam et al., 2018) had a slightly high correlation with one another (the correlation coefficients ranged from 0.66 to 0.79, while in the present study, the correlation coefficients were between 0.702 and 0.827 compared to the original model). In addition, the varimax rotation method applied by Roskam et al. (2018) assumed that the factor axis is orthogonal and that the correlations between factors were 0. With the varimax rotation method, correlations between factors may essentially be underestimated, which means that the correlations between the four factors may have had higher values and, potentially, that these four factors could be combined into three factors or less.

In the present study, the results of the EFA and CFA supported a single-factor structure, and the multi-group CFA supported the gender invariance of factor structure of the PBA, which provides further support for the single-factor structure. The Cronbach’s α showed a consistently high coefficient across gender and time, which suggests a high reliability for the Chinese version of the PBA. A possible reason for the difference of factor structure is that the four factors in the original version were more inherently correlated, and could be combined into a single factor. Another possible explanation is that the cultural differences between the samples caused this dissimilitude in factor structure. People in China and other Asian cultures have a relatively holistic cognitive orientation (Nisbett et al., 2001). Therefore, Chinese parents may consider parental burnout to have a wider scope. In contrast, people in America and other Western cultures may have a more analytic cognitive style (Nisbett et al., 2001), and they may tend to view parental burnout as composed of several different aspects. Further studies should clarify whether the observed differences in the factor structure are caused by the fact that these four factors are actually highly correlated, or because of cultural differences. Furthermore, if the difference in factor structure was caused by cultural differences, it may be necessary to study parental burnout in the Chinese context by applying an inductive approach, as it was applied by Roskam et al. (2018).

In the context of external validity, first, parental burnout is an interpersonal-level variable rather than an individual-level variable. Fathers’ parental burnout showed a positive correlation with mothers’ parental burnout. In addition, because job burnout is closely related to parental burnout, the parents experiencing job burnout may have less enthusiasm to care for their children. Consistent with a prior study (Roskam et al., 2017), parental burnout was found to be positively related to job burnout in emotional exhaustion and depersonalization at both Time 1 and Time 2, which supports the concurrent validity of the Chinese version of the PBA.

Second, the correlation relationship remained significant across time. Parental burnout showed a bidirectional relationship with job burnout. Furthermore, interaction of both fathers’ and mothers’ parental burnout at Time 1 significantly predicted children’s anti-social behavior at Time 2. When both parents showed a high number of burnout symptoms, the children exhibited more anti-social behavior. However, parental burnout generally showed no significant correlations with reduced personal accomplishments. These results were also interpretable because reduced personal accomplishments are not a central component of job burnout, and some studies have shown that reduced personal accomplishment has a different effect on job burnout when compared to that of emotional exhaustion and depersonalization (e.g., Li et al., 2005). Overall, parental burnout showed generally positive correlations with job burnout and children’s anti-social behavior, providing support to the predictive validity of the Chinese version of the PBA.

Third, as noted by Le Vigouroux et al. (2017), personality traits are related to parental burnout. Parents with neuroticism showed greater emotional instability, worried more frequently, and reacted more intensely to life events (Prinzie et al., 2009). Consistent with Le Vigouroux et al. (2017) and Mikolajczak et al. (2018), neuroticism was found to be positively related to parental burnout in this study. Furthermore, previous research has revealed that parents with higher levels of agreeableness made more positive attributions regarding their children’s behavior and were better able to respond to their needs (Prinzie et al., 2009). Consistent with a prior study (Le Vigouroux et al., 2017), agreeableness was found to be positively related to parental burnout, which provided further support to the concurrent validity of the Chinese version of the PBA.

Fourth, both fathers’ and mothers’ parental burnout were positively correlated with children’s life satisfaction at Time 1. Furthermore, mothers’ parental burnout was positively correlated with children’s loneliness at Time 1 and anti-social behavior at Time 2, whereas fathers’ parental burnout was not. These results further supported the concurrent and predictive validity of the Chinese version of the PBA and suggest that mothers play a more important role in parenting events (Starrels, 1994). Future research may consider the parental role in family systems and investigate whether the mother’s role in parenting is more important than the father’s in the Chinese context.

### Limitations and Future Research Directions

Although the present study improved our understanding of parental burnout in China, there are several limitations that should be noted. First, to ensure adequate, longitudinal data collection from family units, our sample was limited to eighth-grade students; consequently, the ages of the sample were relatively concentrated. However, parental burnout is not restricted to the parents of children of a certain age or with certain diagnoses, and the PBA can be utilized across a broad context (Roskam et al., 2017, 2018). Thus, future studies should examine a broader sample with an age range spanning from babies to teenagers.

Second, the main purpose of the present study was to translate the PBA into Chinese and investigate the reliability and validity of this measurement. In particular, we discussed the construct validity, concurrent validity, and predictive validity. Thus, future studies should examine the antecedents and consequences of parental burnout in the Chinese context.

Third, although parental burnout was found to be generally correlated with job burnout, the causal effects remain unclear. Future studies could use a time-lag design to examine the causal effects between parental burnout and job burnout.

## Conclusion

In general, the present study provided preliminary evidence regarding the reliability and validity of the Chinese version of the PBA and provided an effective tool for measuring parental burnout in China.

## Data Availability Statement

The datasets generated for this study are available on request to the corresponding author.

## Ethics Statement

The studies involving human participants were reviewed and approved by the Research Ethics Committee of the Institute of Psychology and Behavior, Henan University. Written informed consent to participate in this study was provided by the participants’ legal guardian/next of kin.

## Author Contributions

All authors listed have made a substantial, direct and intellectual contribution to the work, and approved it for publication.

## Conflict of Interest

The authors declare that the research was conducted in the absence of any commercial or financial relationships that could be construed as a potential conflict of interest.
